# Inadvertent Septal Ablation During Percutaneous Coronary Intervention

**DOI:** 10.7759/cureus.19227

**Published:** 2021-11-03

**Authors:** Feng Gao, Umair Khalid

**Affiliations:** 1 Department of Medicine, Baylor College of Medicine, Houston, USA; 2 Department of Medicine, Michael E. DeBakey Veterans Affairs Medical Center, Houston, USA

**Keywords:** side branch jailing, percutaneous coronary intervention complications, percutaneous coronary intervention, septal ablation, side branch occlusion

## Abstract

We present a case of inadvertent occlusion of a septal artery from being jailed during percutaneous coronary intervention of left anterior descending artery. This resulted in partial loss of the interventricular septum. Risks of side branch occlusion and bifurcation stenting are discussed.

## Introduction

Jailing of nonsignificant side branches during planned percutaneous coronary interventions (PCIs) is not an infrequent complication [1]. This occurs in situations where the proximity of a coronary lesion to an adjacent side branch results in a stent landing site that inevitability occludes the side branch ostium. Such occlusions are majority of the time clinically silent, structurally insignificant, and do not cause conduction defects [1]. Herein, we present a case of side branch occlusion during planned PCI that resulted in an inadvertent septal ablation.

## Case presentation

A 69-year-old man presented to the emergency room with two weeks of exertional dyspnea, unable to ambulate greater than 50 feet. Cardiopulmonary examination showed blood pressure of 131/82 mmHg, heart rate of 77 beats/min, a laterally displaced point of maximal impulse, bilateral dependent lung crackles, and bilateral lower extremity pitting edema. Medical history included uncontrolled hypertension, uncontrolled hyperlipidemia, peripheral artery disease, 50 pack-year smoking history, and alcohol use disorder. No history of recreational drug use was reported. Patient had no prior surgical history. The differential diagnosis for the etiology of clinical heart failure in this patient included ischemic cardiomyopathy, alcoholic cardiomyopathy, hypertensive heart disease, and heart failure with preserved ejection fraction.

**Figure 1:**
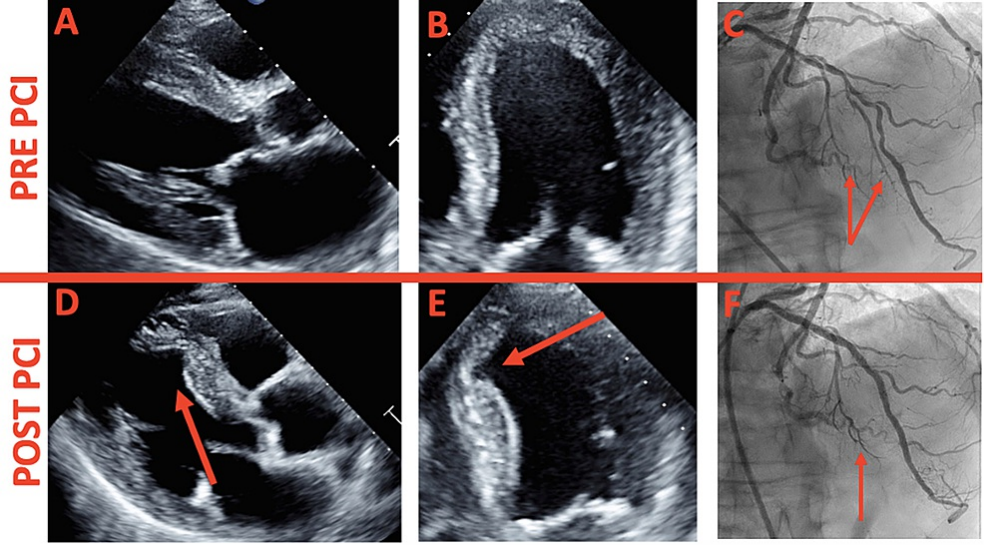
Echocardiography and coronary angiography. (A) Pre-PCI TTE parasternal long view with normal septal thickness. (B) Pre-PCI TTE apical view with normal apical and septal wall thickness. (C) Pre-PCI with the diffuse LAD lesion on coronary angiography measuring 80 mm with ostial stenosis of midseptal branch and diagonal branch (red arrows: two midseptales). (D) Post-PCI TTE parasternal long view with new septal thinning, but no perforation (red arrow: septal defect). (E) Post-PCI TTE apical view with same septal thinning redemonstrated (red arrow: septal defect). (F) Post-PCI of LAD with loss of midseptal branch (red arrow: only one septal left). PCI: percutaneous coronary intervention, TTE: transthoracic echocardiography, LAD: left anterior descending coronary artery.

On admission, a 12-lead electrocardiogram showed sinus rhythm with QS waves in leads V2-V4. Laboratory studies showed troponin I of 0.04 ng/ml and brain natriuretic peptide of 2498 pg/ml. Transthoracic echocardiography (TTE) showed left ventricular ejection fraction of 30%-35% with moderate to severe global hypokinesis. Given patient’s high likelihood of coronary artery disease, he underwent coronary angiography for workup of his cardiomyopathy, which revealed a diffusely diseased left anterior descending coronary artery (LAD) with worst stenosis up to 70%-80% (Figure 1A-1F).

Patient was deemed not a candidate for coronary bypass graft surgery (CABG) by cardiothoracic surgery; thus, PCI with intravascular ultrasound (IVUS) guidance was performed. The LAD lesion was crossed using a Choice PT wire (Boston Scientific, Marlborough, Massachusetts, USA) and predilated using multiple compliant balloons with good expansion. A 3.0 x 28 mm drug-eluting stent (DES) was implanted in the distal LAD, 3.5 x 38 mm DES in the mid-LAD, and 3.5 x 28 mm DES in the proximal LAD with a very good final angiographic result (Figure 1F). The IVUS was used prior to stent implantation for lesion characterization and vessel sizing and post-stenting to confirm adequate stent expansion and apposition without any edge dissection. However, there was an occlusion of a septal perforating artery due to being jailed by the LAD stent struts (Figure 1F). We opted not to intervene on this septal branch to avoid disruption of LAD stent struts. Moreover, the patient did not have any clinical signs or symptoms from losing this septal branch. He did well and was subsequently discharged on appropriate medical therapy.

Six-month post-PCI, TTE showed improved left ventricular ejection fraction to 55%, but thinning of the midanteroseptum and midinferoseptum was noted (Figure 1D-1E). No ventricular septal perforation was detected after evaluating the defect on multiple views with color Doppler.

## Discussion

Side branch occlusion (SBO) may occur in 2%-10% of percutaneous coronary interventions with drug-eluting stents, typically by mechanical straightening of the vessel and shifting of atherosclerotic plaque into side branch ostia [2-4]. Bifurcation stenting confers a higher risk of restenosis; hence, operators often chose provisional approach, especially if the side branch does not supply a large myocardial territory. However, bifurcation stenting is generally not performed for septal perforating arteries. Risk factors for SBO in our case included ostial septal artery stenosis, significant calcification of LAD, and placement of multiple overlapping stents [2].

This case was unique in that the SBO resulted in only partial loss of a septal wall. Such is similar to alcohol septal ablation for hypertrophic cardiomyopathy, although this generally involves ablating the first septal perforator resulting in loss of the basal septum at the left ventricular outflow tract [5]. Luckily the first septal perforator was not occluded in our patient, which would increase risk of complications including pacemaker implant for new heart block (9%-20%) and sustained ventricular arrhythmias (0.7%-14%) [6-8].

As seen in this case, SBO resulting from PCI did have significant septal infarction, even though there were no immediate clinical manifestations. However, if this patient has any LAD territory myocardial infarction (MI) in the future, there is at least a theoretical risk of ventricular septal defect (VSD) from further septal injury. Consequently, all PCI-related SBOs may not be clinically silent and we should take this into consideration prior to jailing any side branch during PCI. This is also one of the potential causes of post-PCI MIs, which we know are related to worse clinical outcomes in the future [9]. More data are needed to examine the clinical impact of PCI-related SBOs. For instance, there have been no studies that compared long-term adverse effects of septal side branch occlusions to occlusion of other side branches. Nonetheless, all prominent side branches should be protected by placing a coronary wire during PCI of main vessel [10,11]. This not only decreases the likelihood of SBO but also serves as a guide to reopen it if necessary.

## Conclusions

Side branch occlusions related to PCI, particularly occlusion of large septal perforating arteries, may not be clinically silent. Risk of side branch occlusion should be taken into consideration when jailing a side branch during main vessel stenting. This risk is especially high in complex bifurcation lesions, presence of multiple layers of stents, and ostial side branch stenosis.
